# The Gendered Politics of Pandemic Relief: Labor and Family Policies in Denmark, Germany, and the United States During COVID-19

**DOI:** 10.1177/00027642211003140

**Published:** 2021-11

**Authors:** Nino Bariola, Caitlyn Collins

**Affiliations:** 1 The University of Texas at Austin, Austin, TX, USA; 2 Washington University in St. Louis, St Louis, MO, USA

**Keywords:** work-family policy, pandemic relief, COVID-19, gender inequality, welfare states

## Abstract

The COVID-19 pandemic has magnified families’ struggles to reconcile caregiving and employment, especially for working mothers. How have different countries reacted to these troubling circumstances? What policies have been implemented to alleviate the pernicious effects of the pandemic on gender and labor inequalities? We examine the policies offered in Denmark, Germany, and the United States, three countries that represent distinct welfare regimes. We find important differences among the policy solutions provided, but also in the “cultural infrastructures” that allow policies to work as intended, or not. In Denmark, a social-democratic welfare state, robust federal salary guarantee programs supplemented an already strong social safety net. The country was among the first to lock down and reorganize health care—and also among the first to reopen schools and child care facilities, acknowledging that parents’ employment depends on child care provisioning, especially for mothers. Germany, a corporatist regime, substantially expanded existing programs and provided generous subsidies. However, despite an ongoing official commitment to reduce gender inequality, the cultural legacy of a father breadwinner/mother caregiver family model meant that reopening child care facilities was not a first priority, which pushed many mothers out of paid work. In the U.S. liberal regime, private organizations—particularly in privileged economic sectors—are the ones primarily offering supports to working parents. Patchwork efforts at lockdown and reopening have meant a lengthy period of limbo for working families, with disastrous consequences for women, especially the most vulnerable. Among such varied “solutions” to the consequences of the pandemic, those of liberal regimes seem to be worsening inequalities. The unprecedented nature of the current pandemic recession suggests a need for scholars to gender the study of economic crises.

Abundant research documents the complications families face as they navigate caregiving and work duties—even before the COVID-19 pandemic upended daily life. Parents in many industrialized countries, for instance, report lower levels of happiness and emotional well-being compared with nonparents (Glass et al., 2016). The struggle is particularly onerous for mothers as they often complete a “second shift” at home after a full day at work (Hochschild, 1989) and because they often have to walk a thin line between cultural expectations of “family devotion” and an ideological mandate to be an “ideal worker” fully committed to their jobs (Blair-Loy, 2003; Williams, 2000). Mothers confront such struggles even in countries where generous policies exist to ameliorate work-family conflict (C. Collins, 2019).

The COVID-19 pandemic intensified these challenges. Scholars are beginning to shed light on some of the ways in which families—and mothers in particular—are carrying the weight. As the pandemic unfolded, mothers with young children in the United States are significantly more likely than fathers to reduce their work hours (C. Collins et al., 2020). For families with young and school-age children, the loss of full-time child care and participation in homeschooling are associated with adverse employment outcomes for mothers, but not for fathers (Petts et al., 2020). In the past few months, mothers exited the labor force to a larger extent than fathers (Landivar et al., 2020). And women workers have been particularly vulnerable during the pandemic: unlike previous economic downturns, the current recession has disproportionately affected women’s jobs, not men’s (Alon et al., 2020). These studies suggest that gender gaps in labor markets are widening in the COVID-19 era. However, scholars have yet to evaluate cross-national variation in policy responses to these vexing circumstances. Previous studies show that mothers’ and parents’ well-being varies by context: they fare better in countries with more progressive, egalitarian work-family policies (Glass et al., 2016). In that spirit, this article explores how different countries are reacting to the harmful effects of the pandemic on gender and labor inequalities. We examine the policies and programs in Denmark, Germany, and the United States, three countries that represent distinct welfare state regimes (Esping-Andersen, 1990).

Considering that the welfare infrastructures of corporatist states like Germany and especially of social democratic states like Denmark include more generous supports than that of liberal states like the United States, we expected countries to follow suit in their respective policy responses to the pandemic. And, indeed, we find that they do: In the United States, the government provided limited support compared with that offered in Germany and Denmark. These two countries already had a robust safety net in place, and they expanded existing programs and protections early in the pandemic to help workers. Hence, the impacts on families and working mothers in particular are mitigated to an extent by state institutions. We also argue that the countries’ *cultural infrastructures*—the systems of state meaning that make institutions work (Norton, 2014)—matter in how these policy responses are designed by the states and experienced by families. As we will see, the policy responses in all three countries are shaped by cultural frames about state-market-family relations and the role of the state in the economy in general and during economic crises.

## Pandemic Policy Responses in Western Welfare States: A Feminist Analysis

As the COVID-19 pandemic upended work and family life, how have different countries reacted to gender and labor inequalities? Evaluating the disparate policy responses various countries implemented is important because it sheds light on contemporary welfare state dynamics in an era of unprecedented economic precarity. Who receives more supports and social protections? Who is left wanting, rendered external to the scope of citizenship and pandemic welfare provisioning? These policy responses are consequential for the lives, livelihoods, and well-being of families and working women in particular.

### A Feminist Approach to Welfare States

Inequality scholars study welfare states because they constitute systems of governmental social provisioning that shape the distribution of resources and opportunities (Pierson, 2000). Welfare states, in that sense, are political interventions in civil society to change social and market forces (Orloff, 1993). Identifying patterns in the principles, policy provisions, and outcomes of these systems, scholars have grouped countries together to facilitate comparisons of various welfare state approaches to social provisioning. The most well-known typology of welfare states groups Western countries into three categories: social democratic, conservative corporatist, and liberal (Esping-Andersen, 1990). *Social democratic states* like those in the Nordic countries of Denmark, Sweden, and Norway assume full responsibility for citizens’ welfare and offer generous governmental policies to support all citizens. These states intervene strongly in the market and family life to promote equality. Their policies support a dual-earner, dual-caregiver family model where both men and women work for pay and care for children. Entitlements are universal and tied to social rights, which strive to protect citizens from market uncertainties and fluctuations to ensure a baseline level of well-being.

*Conservative or corporatist states* such as Germany, France, and Austria take both a regulatory and governmental approach to welfare provisioning. Citizen well-being is considered the responsibility of the government, businesses, communities, and families. Market primacy remains, but the state and employers intervene to support citizens, generally contingent on employment or family position. Historically a man breadwinner/woman caregiver or part-time earner family model has been the norm, bolstered by social policies to support caregiving.

*Liberal states* such as the United States, Britain, Canada, and Australia encourage all citizens to work for pay and turn to the market to meet their needs. The state intervenes little in family life, and when it does, provisions are aimed at the most vulnerable citizens and means-tested according to need. Assistance is left up to individual employers without much state interference. The result is a patchwork of policy supports for working families. Professional, white-collar workers with greater market power (i.e., men, the highly educated, White people) are more likely to have access to policy supports than low-income workers. There exists a consensus among scholars that the free-market approach to family policy has failed U.S. workers, parents, and children (Glass, 2009), and exacerbated inequalities of gender, race, and social class. A dual-earner, woman-caregiver model remains the norm. Few policies support caregiving.

Although scholars debated this categorization for decades, revising and expanding it using different indicators to create new typologies (Amable, 2003; Pierson, 2000), Esping-Andersen’s groupings are still fitting and widely used (e.g., Sachweh, 2019). Thus, bearing in mind the many noteworthy critiques, we use this typology because it remains useful for comparing countries’ distinct approaches with welfare provisioning and social protections at the intersection of state, market, and family relations (Orloff, 1993).

Welfare states may or may not be intended to or produce greater equality among a country’s citizens (Orloff, 1993). A key tenet of the feminist critique of prevailing welfare state theories is that some welfare provisions may support and protect one group at the expense of another, or advance equality in one domain while diminishing it along another (Orloff, 1996). The emphasis on the state-market relationship in conventional welfare state thinking obscured crucial gender dynamics essential to social provisioning:

By focusing only on trade-offs and interplay between states and markets, traditional analysis has obscured and distorted the ways in which state-market interactions depend on, are shaped by, and in turn reconstitute relationships within households, between household and market, and between household and state. (Pierson, 2000, p. 801)

More recent scholarship thus goes beyond considerations of provisions like social insurance and pensions to also consider child care and paid family leave as central features of welfare support. Countries vary widely in the types and effectiveness of their policy supports. A feminist approach is necessary to understand the complexity and nuance of the relationships between states, markets, and families (Orloff, 1993), and to evaluate disparate views of how the costs of social reproduction should be distributed among families, employers, and society at large (Glass, 2009).

We use the conceptual framework of feminist welfare state theory to help make sense of public policy responses to the COVID-19 pandemic. Missing from current conversations about the causes and consequences of gender and labor inequalities surrounding the pandemic is an analysis that explicitly links these disparities to various models of welfare state provisioning. This article begins to fill this gap by comparing pandemic responses in Denmark, Germany, and the United States—three exemplars of Western welfare models.

We seek to extend the welfare state conceptual edifice—including the corresponding feminist critique—in two important ways. First, we use it to theorize by analogy about how conventional policy measures to respond to economic recessions and crises suffer from the lack of a feminist lens that considers and scrutinizes the stratified impacts of downturns. Second, we underscore the important role of cultural mechanisms for the functioning of welfare systems.

### Gendering the Analysis of Economic Downturns

The unprecedented nature of the economic downtown resulting from the COVID-19 pandemic presents an opportunity to reconsider our theorizing of economic crises and recessions. This article posits the need to *gender the scholarly analysis of economic crises*. Historically, economic fluctuations are patterned in somewhat predictable ways, one being that they typically disproportionately impact male-dominated occupations and economic sectors (Alon et al., 2020). Because recessions in the past have mostly affected men’s jobs, researchers tend not to theorize economic crises as gendered (see Cook & Grimshaw, 2020, for an exception). Instead, scholars often take for granted core assumptions about the nature of economic downtowns and neglect their gendered influence on employment—and now, in the pandemic, policy responses from national governments.

Abstracting economic downtowns from gender limits our ability to assess and understand men’s and women’s experiences during recessions, especially in heterosexual couple families as they navigate employment and caregiving (Rao, 2020). The pandemic crisis, though, has seen women suffer the brunt of employment losses (Craig & Churchill, 2020; Hipp & Bünning, 2020; Qian & Fuller, 2020).

We suggest it is time to create theories about economic crises that are not predicated on the concepts of abstract jobs, ideal workers, and absent women. We build on Joan Acker’s (1990) theorizing about organizations as sites of male dominance and her critique of organizational thinking for assuming a disembodied, universal worker to consider how institutions—the economy and economic crises themselves—are deeply gendered. To do so, we apply a feminist perspective to consider the intersection of policy and culture in different countries’ responses to the pandemic and related recession. The economy and the welfare state—especially in corporatist and liberal regimes—are sites of male dominance. This new approach to theorizing economic crises helps us uncover additional ways that welfare states, like organizations, are implicated in shaping gender relations, providing “the subtext for arrangements of subordination” (Acker, 1990, p. 155).

### The Cultural Infrastructure of Welfare States

Recent sociological scholarship suggests the need to deepen the cultural dimensions of institutional analysis of state organizations and processes (Mayrl & Quinn, 2016; Norton, 2014). Institutionalist scholars propose that policies and programs have “interpretive effects” by offering sources of meaning to their beneficiaries (Pierson, 1993). But whereas in classic theorizations policies gave way to meanings, attitudes, and other forms of culture, others suggest a more dynamic, reciprocal relationship (Adams, 2007). In that spirit, we can consider how cultural beliefs and frames are institutionalized in states’ welfare provisioning, and shape culture and social relations (Connell, 1987; Orloff, 1996). And, in turn, welfare states can structure gendered, racialized, and classed “subtexts” regarding social citizenship about who can and should work for pay, who can and should care for family, who can and should receive governmental support (Orloff, 1993).

In this line of theorizing, Norton (2014) calls for the study of “cultural infrastructures,” the systems of state meaning that make institutions work. Whereas previous research about culture and the state focused on practices that state actors undertake to articulate specific beliefs, dispositions, and discourses, this approach centers on “culture as a practical condition of possibility for state action through the mechanism of coordination around public, collective meanings” (Norton, 2014, p. 1544). We thus go beyond considering state policies’ “interpretive effects” to take into account the cultural elements on which policies depend. To achieve their intended effects, policies hinge on a system of meanings that delineates not only who should benefit from them but also how and why the policies should be implemented and used.

We examine the cultural infrastructure of welfare states during the COVID-19 pandemic recession. This includes the cultural cues that provisions generate, the cultural frames that inform people’s expectations as to what the state should do and provide (or not), and information about why the state will offer such provisions and how it will coordinate its delivery or performance. Together, these elements configure a cohesive web of meanings that make policies’ coordination and implementation possible. Dissonance between the system’s different elements may undermine a provision’s efficacy.

## Findings

We summarize relevant background information about Denmark, Germany, and the United States in Table 1, delineating their welfare models, work and parenting cultures, demographics, and family policies. We then examine each of these countries in turn. In each section, we give an overview of the general features of the country’s welfare regime and the government’s response to the pandemic regarding lockdown and health care measures. Then, we examine the relevant labor and fiscal policies implemented and tease out their implications for women, men, and families.

**Table 1.** Demographic Information and Public Policies in Denmark, Germany, and the United States.

	Denmark	Germany	United States
*Political and cultural infrastructure*			
Welfare state model	Social democratic	Corporatist	Liberal
Parenting culture	Dual caregiver	Mother caregiver	Mother caregiver
Work culture	Dual earner	Father earner/mother part-time earner	Dual earner
*Country demographics*			
Population	5.8 Million	83.1 Million	311.6 Million
Employment rate (working-age pop.)	74.0%	75.0%	70.0%
Maternal employment rate^a^	82.0%	69.0%	65.7%
Employed full-time	72.0%	30.0%	53.1%
Employed part-time	9.6%	39.0%	12.4%
Poverty rate	5.8%	10.4%	17.8%
Child poverty rate	3.7%	12.3%	20.9%
*Family policy provisions*			
Public spending, family benefits (% GDP)	3.44%	3.06%	1.12%
Cash	1.36%	1.09%	0.07%
Services	2.08%	1.13%	0.57%
Tax breaks for families	0.0%	0.84%	0.48%
Paid maternity leave	18 Weeks	14 Weeks	0 Weeks
Paid paternity leave	2 Weeks	0 Weeks	0 Weeks
Paid parental leave	32 Weeks	44 Weeks	0 Weeks
Public child care slot	From 6 months	From 1 year	No guarantee

*Source*. Compiled by authors from OECD Family Database <http://www.oecd.org/social/family/database.htm> and OECD Better Life Index <http://www.oecdbetterlifeindex.org/>.

a Maternal employment rates (%) for women (aged 15-64 years) with at least one child aged 0 to 14 years in 2014 or latest available year.

### Denmark

Denmark is renowned for its high metrics of resident well-being. This is explained in large part by Denmark’s social democratic welfare model, which alleviates risk and insecurity, and equalizes opportunities and life chances for citizens. Danes have access to high-quality, free- or low-cost child care, education, and health care. Danish residents report very high levels of trust in the government and society generally (Denmark.dk, 2020). Danes also pay among the highest taxes of people anywhere. The tax-to-GDP ratio was 45.9% in Denmark in 2016, compared with 37.5% in Germany and 26% in the United States (OECD, 2017). Yet Danes report being quite willing to pay high taxes, seeing it as an investment in collective well-being, purchasing quality of life (Wiking, 2016). Gender equality is both a general principle and goal of Danish policy, though women have not reached parity to men on a number of labor force indicators. For example, Denmark ranks lower than other Nordic countries on women in senior leadership positions (Ismiris, 2018).

#### Lockdown and Health Care Measures

Denmark had one of the fastest pandemic responses in all of Europe in mid-March 2020. It rapidly shut its borders, public institutions, schools, and daycares—this even before they were widely accepted or politically savvy measures. On March 13, the Danish government sent home nonessential public sector employees. On March 18, it shut down a wide swath of the private sector. In that 5-day span, the government consulted closely with employer associations (which include half of Danish workers) and trade unions (representing two-thirds of the Danish labor force) to create a comprehensive, mutually agreeable rescue package. These measures also received the support of parties across the political spectrum in the Danish parliament. By showing a united front and a sense of mutual responsibility for the lockdown, the Danish government helped secure widespread public support and compliance, which we now know is key to reducing infection rates (Ornston, 2020). Public compliance was made possible in part because, before the pandemic, Danes had strong interpersonal trust, confidence in public expertise, and trust in public authorities. This also meant Danish society was better able to leave lockdown and reopen in mid to late April. Their fast actions were effective. Cases of COVID-19 dropped markedly and fast, remaining low until December, when rates spiked again and the government took the same approach for a second lockdown.

Denmark’s strong public health care system was also able to pivot quickly to meet the needs of Danes. The entire system—already free for all, regardless of health insurance—adjusted by reorganizing and reprioritizing departments, isolation wards, hospital beds, doctors, and nurses in order to treat and admit ailing patients (Olagnier & Mogensen, 2020). The state also partnered with Novo Nordisk, a large Danish pharmaceutical company in early April, which allowed Denmark to rapidly increase testing as they reopened at one of the highest rates globally.

#### Labor and Fiscal Measures

**Figure 1.**
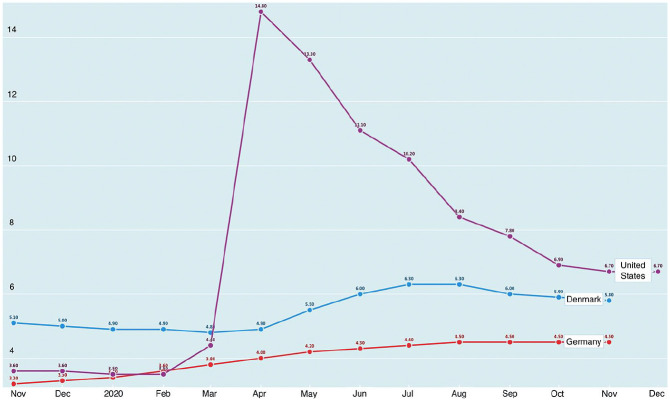
Monthly unemployment rates in Denmark, Germany, and the United States, November 2019-December 2020. *Note*. Cross-national data disaggregated by parental status are unavailable from OECD or Eurostat. *Source*. OECD data compiled at https://data.oecd.org/

In response to the pandemic, the Danish government implemented what has been called one of the most dramatic, far-reaching economic plans in the world (Thompson, 2020). *The Atlantic* economics writer Derek Thompson described it as a “radical idea [ . . . ] unlike anything I’ve ever heard” (Thompson, 2020)—this in an article titled, “‘Do More—Fast. Don’t Wait.’ Denmark, Which is Basically Freezing the Economy, Has a Message for America.” The state covered the costs of up to 90% of wages for private employees who remained on payroll but went home and stopped working. This discouraged mass layoffs and helped explain Denmark’s 4.9% unemployment rate in April. At the same time, this figure was 14.8% in the United States and 4% in Germany (see Figure 1).

Danes already have a strong social safety net. Rather than expanding their unemployment program to strengthen this safety net, the Danish government took the unprecedented step of paying private businesses not to fire employees. Freelancers and self-employed workers were eligible for up to 75% of their expected income lost during the lockdown. Public sector employees were kept on and permitted to work from home with no salary consequences.

The rescue package included other economic measures, too. It provided funding to cover fixed costs for businesses like rent, leasing expenses, and interest rate expenses to help them stay afloat during the lockdown. And employers and self-employed people were permitted to postpone paying taxes and entitlements to sickness benefits. While employers are typically responsible for the first 30 days of paid sick leave for employees, the government took over these payments from day one for workers who fell ill from the coronavirus.

The price tag for this plan was massive—up to 13% of GDP in a mere 3 months. But the Danish government recognized that high unemployment and reduced aggregate demand would cost far more to the economy and the national deficit than that amount in the months to follow. Said Denmark’s employment minister Peter Hummelgaard, “That’s the economic side of it. It is more expensive to do less. Then there’s a social side of it: Unemployment creates a host of problems not only for society, but also for individuals” (Thompson, 2020).

Even before the pandemic, Denmark already understood this reality, and had an active labor market policy model called “flexicurity.” This model combines labor market flexibility in a dynamic economy with security for workers. It is meant to limit financial risk to both employers and employees (Wiking, 2016). Flexicurity means that Danish employers may be more willing to take a chance on a potential employee, and workers feel more comfortable changing jobs in order to advance. Roughly one in four Danes working in private industry change jobs each year (Denmark.dk, 2020). The extreme measures undertaken to freeze the economy during the pandemic were in part a response to this flexicurity model, and their passage seems to have saved the country from a massive recession and high rates of unemployment.

#### Mitigating Gender Disparities?

Besides its pandemic labor and economic policies, another major reason that Denmark was able to close and reopen so quickly in spring 2020 is its universal child care system. For Danes with young children, everyday life in nonpandemic times already entails significant financial and practical support from the Danish government. Public spending on family benefits is roughly 4% of GDP, and 60% of that spending is for family services, including child care. Children are entitled to a place in public child care from age 6 months onward. Denmark offers public preschool starting at age 3 years. State-run care is also available outside of school hours. Over half of children (55.4%) aged 0 to 2 years attend public child care, as do 97.5% of children aged 3 to 5 years. When it was decided that day cares and schools should close down, the government was able to mandate and enact this swiftly. Countries without a centralized public child care system have not been able to carry out or oversee such widespread safety measures for children and families.

As the pandemic unfolded, Denmark was also the first to relax restrictions on child care and education as the country reopened after a month of lockdown. This move allowed children up to 11 years old to return to facilities that had instituted a host of new safety measures on April 15 (“Coronavirus: Denmark Lets Young Children Return to School,” 2020). Although facilities couldn’t enroll the same number of children as before given the social distancing guidelines, and some parents opted to keep their children home due to safety concerns, this strategic reopening freed up many of the nation’s parents to return to work (Elabdi, 2020). The lockdown in December 2020-January 2021 also prioritized public care for young school-age children, allowing students up to fourth grade to continue face-to-face schooling (when children need the most intensive adult supervision), with older students learning virtually, for all but 2 weeks when all schools closed at the peak of the second spike.

These moves meant that the crunch period for lots of working parents—managing around-the-clock caregiving with simultaneous remote work—was short-lived, at roughly a month in the spring and several weeks in the winter. This approach stood in stark contrast to countries like the United States, where this difficult scenario has stretched on for many months, with devastating consequences for mothers’ labor force attachment. In this way, Denmark’s public child care system facilitated parents’ rapid return to work, especially for mothers, who despite strides toward gender equality, still do more of the caregiving than do men. By effectively shortening the windows in which families lived in limbo at home, working for pay while somehow also caring for their children, the Danish government was thus able to ameliorate some of the worst gendered consequences of the pandemic. And, of course, returning to work “full-time” looks different in Denmark than in many other western industrialized countries: The standard full-time work week is 37 hours, while most people work 30 to 35 hours a week, women and men alike (OECD, 2018).

The rescue package also helped prevent layoffs that may have disproportionately affected mothers, as was the case in the United States (Landivar et al., 2020). It gave both mothers and fathers the ability to be home with children. However, some worried that it was mothers who took on more of the added caregiving during the lockdown. Wrote one Danish working mother in an editorial in the national newspaper *Berlingske* in late April, families have “gone back to old-fashioned gender roles: Fathers primarily maintained their full-time jobs, while mothers primarily took care of the children” (Madsen, 2020). News reports and studies made similar observations in countries ranging from the United Kingdom (Ascher, 2020; Ferguson, 2020) to Iceland (Hjálmsdóttir & Bjarnadóttir, 2020), a nation renowned for topping gender equality indices globally year after year.

However, two points suggest that this return to “old-fashioned gender roles” was perhaps milder in social democratic countries like Denmark than in Germany or the United States: first, the closure of schools and child care facilities was shorter in Denmark, so the period in which women took on a greater share of domestic work was also likely shorter and therefore less consequential in its long-term effects. And second, households in Denmark were already more egalitarian in the time spent in unpaid domestic work than those in Germany and the United States. Recent time use data before the pandemic show that the ratio of men’s to women’s time spent in domestic work was 77% in Denmark, compared with 62% in Germany and 60% in the United States (OECD, 2020b). So a turn to more traditional roles may very well mean they still rank more equally in the gender division of household labor than in these other countries with woman-caregiver parenting cultures.

### Germany

Germany is often regarded as the prototypical conservative-corporatist welfare regime (Leitner & Lessenich, 2003). In that sense, most entitlements—including health insurance and pensions—are employment-based, and different programs are available for different occupational groups (Esping-Andersen, 1999). The country had for years embraced and promoted a traditional man breadwinner/woman caregiver family model (Rosenfeld et al., 2004) as part of a “subsidiary” welfare model, according to which the family is the preeminent provider of social welfare (Esping-Andersen, 1990). However, scholars argue that policy reforms in recent years are transforming the conservative-corporatist character of the German state. These reforms include some degree of flexibilization of the labor market (Scruggs & Allan, 2008) and the enactment of more gender-egalitarian supports for families (C. Collins, 2019).

#### Lockdown and Health Care Measures

During the first months of the pandemic, Germany’s reaction to the pandemic was widely praised by American and European media outlets (e.g., Bennhold & Eddy, 2020; “Germany Tears Up Fiscal Rule Book,” 2020) and the German citizenry alike (Pew Research Center, 2020b). Germany’s political response to the pandemic was deemed clear and effective, unlike the mixed messages that the U.S. President and other authorities there offered. North American journalists even referred to it as a “masterclass in science communication,” highlighting the fact that Chancellor Angela Merkel is a scientist herself (Farr, 2020). The “radical measures” delineated by the central government on March 16 included a firm nationwide lockdown and closure of most public institutions—including schools and daycares—extensive and consistent testing, and a “track and trace” system to reconstruct “all the chains of infection and interrupt them,” as Jens Spahn, Germany’s Health Minister, indicated (Chazan, 2020).

One important factor for Germany’s successful management of the first few months of the crisis has to do with the country’s robust health care system. As part of a strong safety net, a solid health care system has always been a priority in Germany (Oduncu, 2013). Before the pandemic, Germany had about 28,000 intensive care beds, and they expanded to 40,000 in just a few months. There was no reported shortage of intensive care beds until December, when according to news reports only about 10% were available.

Enacting far-reaching policies in a country with Germany’s federalist institutional structure could prove to be a significant challenge because the Constitution provides state (*Länder*) authorities and local mayors considerable power and responsibility. But Chancellor Merkel insisted on the need to coordinate swiftly and closely with state and local leaders to guarantee the legitimacy of the measures. Along these lines, as in Denmark, several commentators point to leadership and efficiency as key factors in dealing with the pandemic crisis (Stelzenmüller & Denney, 2020). The German citizenry largely trusted the government’s response and complied with the lockdown measures from the outset. A Pew Research Center (2020b) report indicates that 88% of Germans—compared with 95% of Danes and only 47% of U.S. residents (almost exclusively conservative)—believe that their government handled the pandemic correctly. The adequate management of the pandemic allowed Merkel to fashion herself as one of the most popular leaders in the world and the most cherished in Germany.

However, as weeks passed and the lockdown proved effective to manage and reduce the number of COVID-19 cases, support for some of the lockdown measures declined (Naumann et al., 2020). On May 6—after almost 2 months of lockdown—Chancellor Merkel announced a controlled restart of public life: “We can afford a little audacity,” she said. Restaurants, stores, schools, and other public facilities could open following safety measures defined by local governments. In October, as cases rose once again, the government imposed a “light lockdown,” closing restaurants, bars, and cinemas but keeping schools, hotels, and nonessential stores open and allowing meetings of small groups of people. These measures were unsuccessful at containing the spread of the virus (Eddy, 2020). By late December, despite the government imposing a stricter lockdown and closing schools and nonessential stores, Germany reached a record number of deaths, surpassing 30,000.

#### Labor and Fiscal Measures

Aside from the lockdown and health care measures, Germany’s labor and economic relief policies were quite radical. The government, in the words of Constanze Stelzenmüller and Sam Denney (2020) of the Brookings Institute, “tore up its fiscal rule book with the most comprehensive package of salvage measures in Europe.” The federal authorities devised a massive €700 billion plan—that is, the equivalent of two annual national budgets— for liquid guarantees for larger firms, and grants for small companies and freelancers. But perhaps the most potent measure to battle against the recessionary effects of the pandemic was the *Kurzarbeit* program.

*Kurzarbeit* is a crisis management tool that protects workers’ income and supports aggregate demand. It is a social insurance program in which employers reduce employees’ working hours instead of laying them off. The government pays workers at least 60%—67% for working parents—of their regular pay for the hours not worked (International Monetary Fund, 2020). Before the pandemic, 75% of the working-age population had a paid job. Unemployment had been reduced to half of what it was a decade prior (Audretsch & Lehmann, 2016), and even if workers were to become unemployed, they suffer only a 2.7% loss of earnings, which is substantially less than the OECD average of 7% (OECD, 2020a). Years of fiscal prudence had prepared Germany in institutional and financial terms to confront a downturn. Indeed, the fact that Germany was well equipped to handle a crisis was put to the test during the Great Recession—certainly better equipped than liberal welfare states, like the United Kingdom and the United States, and even some social democratic ones, like Sweden (Sachweh, 2019). Considering that Germans regard job stability as a fundamental aspect of their political economy (Kiess et al., 2017), the political goal at the core of the country’s response to the Great Recession was to keep workers in their jobs (Chung & Thewissen, 2011). Among the policies leveraged for this purpose, one of the most effective was the *Kurzarbeit* (Sachweh, 2019). This program enabled 3.3 million workers to keep their jobs during the crisis of 2009.

During the COVID-19 pandemic, the central government substantially expanded funds for *Kurzarbeit*, expecting even more applications than during the Great Recession. Indeed, by May, 2020, more than 10 million Germans had requested *Kurzarbeit*. The expansion of this benefit, among other measures, allowed Germany to keep the unemployment rate around 4% (see Figure 1).

#### Mitigating Gender Disparities?

Despite having some gender-sensitive features—that is, extra money for families and the government’s decision to include temporary workers as potential beneficiaries since the start of the pandemic—it is possible that *Kurzarbeit* may also be having some stratifying impacts in terms of gender. The program, for instance, does not include workers that perform “mini jobs,” a form of marginal, temporary employment that garners particularly low wages. About 28% of working women in Germany perform labor that falls within this category (Cook & Grimshaw, 2020).

Arguably the most troubling measure the German government undertook has been the prolonged closure of schools and child care facilities. Such policy, while crucial to contain the spread of the virus at the onset of the crisis, entailed pernicious effects for women’s labor conditions and job prospects (Hipp & Bünning, 2020; Reichelt et al., 2020). To be sure, schools reopened in mid-May after 2 months of closure. However, for a few months since, many schools only admitted students from older grades in order for them to graduate (Naumann et al., 2020). Younger children remained at home under the care of their parents—mostly their mothers.

Despite recent policy reforms toward more egalitarian gender relations, a myriad of disparities remain in Germany. The working hours gap among men and women is still high, as is occupational sex segregation (Dämmrich & Blossfeld, 2020). A traditional gendered division of labor between household and professional labor prevails. Moreover, the institutional legacies of a “subsidiary” welfare regime imply that the consideration of mothers as the main source of care remains a sort of cultural substratum: Whenever care is needed, women should be the first to provide it. Indeed, research before the pandemic shows that German women do roughly 100 minutes more household labor per day than men (Altintas & Sullivan, 2016). Some scholars are beginning to empirically demonstrate that the pandemic has strengthened these gendered patterns. Hipp and Bünning (2020) reveal that “mothers had a higher likelihood than fathers of working less, and this likelihood increased over time (from 4 to 7 percentage points . . . ).” As in Denmark, in the words of a German entrepreneur, “Women are paying the price for society” in the COVID-19 pandemic (Schmidt, 2020).

### The United States

Experts agree that the effects of the COVID-19 pandemic in the United States—which include almost 400,000 deaths by the end of 2020 and an unemployment rate that ranged from 14.7% in April to 6.7% in December—could have been less disastrous with stronger national leadership and coordination (e.g., Redlener et al., 2020). While we certainly concur with this assessment, we argue that other longstanding institutional and cultural features bounded the country to devastating outcomes, particularly for families and working mothers (Heggeness, 2020; Landivar et al., 2020).

Among the liberal welfare states, the United States is arguably the most radical in its recourse to the market as the main distributional means for addressing basic needs (Gornick & Meyers, 2003). In that sense, of all the OECD nations, the United States is unique insofar it has no federal policy for providing—directly or through employers—all families with significant supports to navigate care and job responsibilities, nor any type of paid sick leave system. The country also lacks universal health care and universal child care. There are limited means-tested provisions of health care and child care targeted for the poorest citizens. The one existing national leave policy—the Family and Medical Leave Act—offers only 12 weeks of unpaid leave, although with no assurance that a worker would get the very same position she held before taking the leave. Moreover, it does not apply to all workers. Although a few states provide more generous benefits, private organizations are increasingly the ones offering more substantive supports in response to families’ incrementing sensation of stress. This creates and reinforces inequalities: Only workers in certain organizations in privileged sectors and industries have the ability to secure such supports (Pew Research Center, 2020a).

#### Lockdown and Health Care Measures

Besides confusing communications that disregarded the deathly character of the virus and the urgency of the crisis, the federal government provided generic, sometimes even questionable guidelines to state and local authorities (Goodnough, 2020). States and cities enacted lockdown measures of varied strictness depending on evaluations of the number of cases and the crisis’ effects on local health care provision. However, such evaluations were at times met with problematic moralized logics that pitted the health of the community against the “health of the economy,” sometimes privileging the latter. Lockdown orders, and the public’s compliance, have therefore been patchwork at best.

The U.S. health care system already suffered “deep underlying problems” even before the COVID-19 pandemic, and the crisis only magnified such problems (Blumenthal et al., 2020). The lack of universal health care and the continued reliance on employer-sponsored private insurance meant that as millions of U.S. Americans lost their jobs, they also lost their health insurance coverage. An Urban Institute report estimates that 10.1 million U.S. Americans would lose employer coverage (Banthin et al., 2020). The same report indicates that, although many of them became eligible for Medicaid or affordable private plans once becoming unemployed, at least 3.5 million people would be uninsured.

A second structural problem of the health care system intensified by the COVID-19 crisis was its historical inability to provide adequate and equitable care to communities of color. Not only are African Americans and Latinxs less likely to be insured, but they are also more likely to suffer from chronic illnesses that can render COVID-19 deadlier (Blumenthal et al, 2020). People of color are also more likely to hold precarious and riskier jobs that increase their exposure risk to the virus (Dubay et al., 2020). The outcome of this is that Latinxs and African Americans are 2.8 times more likely to die of COVID-19 than White U.S. Americans (Centers for Disease Control and Prevention [CDC], 2020).

#### Labor and Fiscal Measures

The United States passed a series of policy packages with bipartisan support to provide temporary benefits and protections to citizens, businesses, and corporations throughout the first few months of the pandemic. These included an expansion of unemployment insurance, a paid leave program, aid to small businesses, bailouts to major corporations, and a one-time direct cash payment of $1200 per adult person in a household with an extra $500 for every child.^1^ But it is in *how* these measures were implemented, understood, and experienced by policy makers and their intended beneficiaries that the structural and cultural characteristics mentioned throughout this section became palpable, limiting their impact and reach, and widening preexisting social inequalities.

Roughly $800 billion were allocated to expand the unemployment benefits of Americans that lost their jobs involuntarily due to the pandemic. By April, the U.S. economy had lost more than 22 million jobs, and only about half of those have been gained back in the months since (Brown, 2020). Although some states are more generous than others, unemployed workers received approximately 45% of their lost income. The federal government provided a temporary payment of $600 per week until July 31. This new policy included furloughed, quarantined, and partially unemployed folks, who were not considered a potential target audience for such support before the COVID-19 era (Goger et al., 2020). Many U.S. Americans benefited from this mandate, and according to experts, the policy has been essential to keep consumption demand up (Rugaber & D’Innocenzio, 2020). However, the federal funds dispensed to support the program ran out at the end of July. Once these funds ran out, the legislative process for a new law to continue providing unemployment benefits got entangled in and stymied by partisan disagreements. These bitter debates included recourses to the racialized tropes of undeservingness and the empirically false, time-old adage that unemployment supports disincentivize engagement in paid work (Goger et al., 2020). Furthermore, unlike *Kurzarbeit* in Germany and the analogous policies in Denmark—for which employers applied directly to the government on behalf of workers without the need to actually lay anyone off—in the United States, people had to apply for benefits themselves once they were fired or furloughed.^2^ Compared with Germans and Danes, who remained contractually tied to their employers for the time being, U.S. residents had to suffer the social implications of unemployment: increased stress, uncertainty, lack of health care, and so on (Rao, 2020).

Moreover, because millions of Americans were applying at the same time, the bureaucratic process often demanded titanic endurance and patience (and even some luck) from applicants. People faced a lack of clear instructions about eligibility and the information needed to apply, remarkably long waits on the phone, and recurrent crashes on the states’ websites where applications should be submitted. A mother shared her experience applying for unemployment to a news outlet, indicating that even as a professional bookkeeper, she felt inept at fully understanding and navigating California’s system: “Nothing was made clear. . . . There was a lot of contradictory information online” (Lozano, 2020). Another report profiles a Maryland woman who spent more than 4 hours on the phone to reach someone: “You can’t do anything online—you can’t get through to anybody . . . I give myself breaks, or I’ll go insane” (MacGillis, 2020). The system disputed her eligibility and, ultimately she was unable to get benefits: “I’m stuck in this position where I can’t get unemployment at all” (MacGillis, 2020).

#### Mitigating Gender Disparities?

To understand the federal government’s approach to pandemic relief for workers and families first requires understanding that the United States is also singular for the salience of a cultural frame of personal responsibility, which limits the capacity of U.S. residents to envision caregiving as a public issue (Dalley, 1988; Levitsky, 2014). Historically, U.S. Americans have placed the duty of care on themselves as individuals or singular families (Dalley, 1988). While some experts expected that the notable increase of women into the labor force would push the lack of a national infrastructure for care to the brink of a crisis that would bring about institutional change (Hochschild, 1983), U.S. Americans continue to individualize what is, in essence, a collective issue, even in the face of insurmountable work-family conflict (C. Collins, 2019; Gornick & Meyers, 2003). Unlike in Denmark and Germany, where families and working mothers perceive welfare provisions as rights, in the United States, they conceive of them as privileges that they can earn through hard work or out of the goodwill of their employers (C. Collins, 2020). The country’s free-market approach, coupled with the cultural ideology of personal responsibility, mean that those with greatest access to work-family policy supports like paid parental leave, health care, schedule flexibility, and paid vacation and sick days are those who already occupy the most privileged positions: workers who are White, wealthy, and men (C. Collins, 2019).

The ideology of personal responsibility deepens existing gender, class, and racial inequalities. As Sandra Levitsky writes, “the assumption that family members should take care of their own is really an assumption that *women* should take care of their own” (2014, p. 7). Indeed, before the pandemic, women were the ones to carry the largest share of the burden for care. Married mothers average 1.9 times the chores and child care of married fathers in the United States (Bianchi et al., 2012). Since most women today work for pay (U.S. Bureau of Labor Statistics, 2020), they tend to feel “competing devotions” between the cultural mandate of limitless work commitment and the ideology that women should prioritize their families above all else (Blair-Loy, 2003). Furthermore, the lack of a public care infrastructure disproportionately affects low-income parents and people of color as they often lack the financial resources to afford quality help (Levitsky, 2014). Finally, the ideology of personal responsibility is also tied to a racialized and heavily pejorative conception of “welfare” as government handouts oriented to an alleged “undeserving class” and as aids that disincentivize the engagement of the poor with paid labor (J. L. Collins & Mayer, 2010; Soss, 2005). This stigmatized perception of welfare not only undermines the efficacy of the programs—as their beneficiaries often have to deal with tradeoffs between getting the benefits and being publicly humiliated for their use—but it also negatively affects public support for the policies from other sectors of society (Brady & Bostic, 2015; Soss, 2005).

These cultural and institutional features have undergirded the country’s response to the pandemic. The paid leave program created during the pandemic was inadequate and patchwork at best. It offered 2 weeks of paid sick leave for workers of small- and medium-sized companies that had been employed for at least 30 days prior. Part-time workers—including gig workers—and self-employed folks were also eligible for this benefit. Some workers could get a special form of paid leave of up to two thirds of their usual pay for 12 weeks to care for children whose schools or child care facilities were closed because of the pandemic. While such benefits are indeed limited compared with those offered in Denmark and Germany even before the pandemic, they *could* have been helpful for far many more families and working mothers than they actually were.

But few people have taken advantage of the federal paid leave program. Why? One issue was that the program received remarkably little publicity. According to *The New York Times* survey, by May, about half of U.S. Americans had heard very little or nothing at all about the benefit and only 13% said they were properly informed about it (Miller & Tankersley, 2020). In addition, a widely shared perception was that paid leave policies such as this could potentially harm businesses. A report by the Bipartisan Policy Center (2020) suggests that 37% of small business leaders still believe—even amidst the pandemic—such policy could hurt their businesses. The limited use of this program should perhaps not be too surprising given the weak institutional character of leave policies in the country, which puts their implementation at the discretion of managers at the level of individual organizations (C. Collins, 2019; Gornick & Meyers, 2003). Add to this the fact that U.S. mothers share a cultural frame that the ability to take leave is a privilege—not a right—and a matter of luck (C. Collins, 2019, 2020).

Arguably the issue that affected U.S. working mothers the most during the pandemic has been the lack of child care availability. Schools and many day cares in all 50 states remained closed from March until late September or October. The debate about reopening schools got entangled in partisan politics, with several Democratic congressmen demanding that schools remain closed until sufficient safeguards were in place to protect teachers, while Republicans pushed for reopening. The latter argument dismissed the deadly character of COVID-19, embracing the logic that the economy comes first. This same logic explained why throughout the summer months several states opted to open bars, restaurants, movie theaters, and other establishments, which later caused surges of the virus and hindered the ability of local schools to plan a safe reopening. In mid-July, the CDC issued safety recommendations for schools, which were then challenged and “edited” by the White House (Meckler & Weiner, 2020). According to several experts, while reopening schools for children is critically important, the safety measures to do so effectively without generating another outbreak should be extremely cautious and follow scientific evidence (Goodnough, 2020). The official guidelines issued by the CDC fell short in that regard, downplaying the risks (Meckler & Weiner, 2020) and leading to confusion among parents (Strauss, 2020). Many parents reacted to these circumstances by opting for “remote learning” or developing solutions of their own: Some moved their kids to private schools with smaller enrollments. Others took their children out of the formal school system and began homeschooling. Many families who could afford it also hired private au pairs and nannies.

The privatization of social problems, as mentioned above, is a characteristic cultural feature of the U.S. liberal welfare regime. But as working parents take on the added roles of teacher, sports coach, and entertainer because of the pandemic lockdown, their stress has skyrocketed—again, especially for mothers (Forde, 2020; Kitchener, 2020). Private companies like Google, Microsoft, and Apple have reacted to this situation by offering some solutions that range from subsidized care (i.e., pay for nannies and au pairs) to online camps for kids and the ability to take several weeks of paid leave or to reduce working hours. While several of these corporations offered generous benefits before the pandemic, they extended or improved their options in light of the lockdowns. Facebook, for example, provides 10 weeks of paid leave and 20 days of fully paid care (Miller, 2020). However, the large majority of U.S. workers lack access to such alternatives. Access is strongly stratified by socioeconomic status (Miller, 2020).

These dynamics altogether are widening preexisting disparities. Women and working mothers continue to take the larger toll compared with men and fathers. Research suggests that mothers of young children, in particular, have suffered the deepest impact in terms of work hours reductions and labor force exits (C. Collins et al., 2020; Landivar et al., 2020). And while the country’s weakened child care system has hurt all families, the effects are especially harmful to families of color. Longstanding structural inequalities mean that parents of color have less access to flexible schedules and telework options to accommodate caregiving than white parents, and women of color are disproportionately represented among essential frontline workers, putting them at increased risk of infection and limiting the time available to care for children (Novoa, 2020). Single mothers—who are disproportionately racial and ethnic minorities in the United States—rely on daycare and extended networks to care for children, but now too often find themselves cut off from these crucial supports (Powell, 2020). Furthermore, low-income families already struggling to make ends meet before the pandemic are in even worse straits now: food insecurity has tripled among households with children. Black and Hispanic families are twice as likely to experience food insecurity as White families in the pandemic (Schanzenbach & Pitts, 2020). In the meantime, during the crisis, billionaire families’ wealth has soared (Reuters Staff, 2020).

## Discussion

Whereas the scholarship is growing on the grim social and economic consequences of the COVID-19 crisis for mothers and families, little research has explored such effects from a comparative standpoint. Considering the institutional frameworks for social protection that configure the different types of welfare regimes (Esping-Andersen, 1999), this article examined the varied reactions and relief policies that three countries—Denmark, Germany, and the United States—devised and executed to alleviate the daunting implications of the pandemic for workers and their families. We find important cross-national variation in the design and implementation of pandemic relief. Such variation is consistent, to an extent, with the countries’ respective welfare regimes. Countries like Denmark and Germany—because of their institutional features as, respectively, a social democratic welfare regime and a conservative-corporatist one, had strong social safety nets and consistent, efficient employment protection policies already in place. During the pandemic, citizens of these countries were already better supported, and families, who otherwise would have fared far worse, were covered by benefits and protections to an extent. In the United States, we see the consequences of lack of a safety net in the dramatically increased inequalities of gender, race, and class.

However, an important contribution of this paper points to the crucial role of “cultural infrastructures” in the policy process as well. Cultural infrastructures—the systems of cultural meanings that allow institutions to work (or not; Norton, 2014)—delineate how policies operate in practice, in the “real world,” beyond the intended effects articulated in plans and legal instruments. Each countries’ cultural infrastructure provides one explanation to the gendered political nature of pandemic relief. Why is it that in a relatively generous corporatist nation like Germany, has the pandemic, as one working mother said, “set gender roles back a few decades” (Schmidt, 2020)? Despite the recent efforts toward more gender-egalitarianism, the legacy of a man breadwinner/woman caregiver familial model was borne out during the pandemic as mothers mostly took on added caregiving responsibilities. In Denmark, in comparison, where a gender-egalitarian culture is more widespread, reopening daycares and schools for younger children was deemed a first priority. In the United States, the prevalence of the cultural frame of personal responsibility, and in general, the tendency to individualize social issues, affected the management of the pandemic in ways that were particularly harmful to women’s and working mothers’ economic prospects.

Another vital aspect of the gendered politics of pandemic relief has to do with the need to scrutinize crises like the one generated by the COVID-19 pandemic from a feminist vantage point. Given that past economic recessions harmed men’s employment the most, scholars have not analyzed these downtowns through the lens of gender. But the current pandemic recession has primarily affected women’s employment across the globe, both in terms of supply and demand. Most jobs lost were in sectors dominated by women (the service sector, including retail, hospitality, restaurants, travel, education, and care), and the shuttering of daycares and schools put unparalleled stress on households, which women largely shouldered (Alon et al., 2020). Women’s disproportionate association with and responsibility for care means they have taken a far bigger hit than men in the current pandemic recession. Because the current recession has harmed women more than men in an unprecedented way, especially in the United States where the pandemic has dragged on for months, some scholars are newly realizing that gender is central to the workings and consequences of economic crises. It is time to theorize them as such.

## Notes

1. In an unprecedented move, stimulus checks were printed with “President Donald J. Trump” in the memo line in the left corner. This was the first time a U.S. president’s name has ever appeared on a disbursement from the Internal Revenue Service—a last minute move at Trump’s urging that may have delayed the mailing of checks to millions of families (Rein, 2020).

2. Since the Great Recession, a few states developed workshare programs similar to *Kurzarbeit*. Such programs, however, have not been widely publicized and only a very small fraction of employers chose to use them (MacGillis, 2020).

## References

[bibr1-00027642211003140] Acker J. (1990). Hierarchies, jobs, bodies: A theory of gendered organizations. Gender & Society, 4(2), 139-158. 10.1177/089124390004002002

[bibr2-00027642211003140] Adams J. (2007). The familial state: Ruling families and merchant capitalism in early modern Europe. Cornell University Press.

[bibr3-00027642211003140] Alon T. Doepke M. Olmstead-Rumsey J. Tertilt M. (2020). This time it’s different: The role of women’s employment in a pandemic recession. National Bureau of Economic Research. 10.3386/w27660

[bibr4-00027642211003140] Amable B. (2003). The diversity of modern capitalism. Oxford University Press.

[bibr5-00027642211003140] Altintas E. Sullivan O. (2016). Fifty years of change updated: Cross-national gender convergence in housework. Demographic Research, 35(July-December), 455-470. http://www.jstor.org/stable/26332084

[bibr6-00027642211003140] Ascher D. (2020, May 27). Coronavirus: Mums do most childcare and chores in lockdown. BBC News. https://www.bbc.com/news/business-52808930?intlink_from_url=https://www.bbc.com/news/topics/c77jz3mdqq1t/motherhood&

[bibr7-00027642211003140] Audretsch D. B. Lehmann E. (2016). The seven secrets of Germany: Economic resilience in an era of global turbulence. Oxford University Press.

[bibr8-00027642211003140] Banthin J. Simpson M. Buettgens M. Blumberg L. J. Wang R. (2020). Changes in health insurance coverage due to the COVID-19 recession: Preliminary estimates using microsimulation. Urban Institute. https://www.urban.org/research/publication/changes-health-insurance-coverage-due-covid-19-recession

[bibr9-00027642211003140] Bennhold K. Eddy M. (2020, May 6). Germany’s reopening offers hope for a semblance of normal life. The New York Times. https://www.nytimes.com/2020/05/06/world/europe/germany-merkel-coronavirus-reopening.html

[bibr10-00027642211003140] Bianchi S. M. Sayer L. C. Milkie M. A. Robinson J. P. (2012). Housework: Who did, does or will do it, and how much does it matter? Social Forces, 91(1), 55-63. 10.1093/sf/sos120 PMC4242525 25429165

[bibr11-00027642211003140] Bipartisan Policy Center. (2020). New BPC/morning consult survey of small business owners: Family and child responsibilities are top challenges during COVID-19. Bipartisan Policy Center. https://bipartisanpolicy.org/blog/new-bpc-morning-consult-survey-of-small-business-owners-family-and-child-responsibilities-are-top-challenge-during-covid-19/

[bibr12-00027642211003140] Blair-Loy M. (2003). Competing devotions: Career and family among women executives. Harvard University Press.

[bibr13-00027642211003140] Blumenthal D. Fowler E. J. Abrams M. Collins S. R. (2020). Covid-19: Implications for the Health Care System. New England Journal of Medicine, 383(15), 1483-1488. 10.1056/NEJMsb2021088 32706956

[bibr14-00027642211003140] Brady D. Bostic A. (2015). Paradoxes of social policy: Welfare transfers, relative poverty, and redistribution preferences. American Sociological Review, 80(2), 268-298. 10.1177/0003122415573049

[bibr15-00027642211003140] Brown S. (2020, July 1). The COVID-19 crisis continues to have uneven economic impact by race and ethnicity. Urban Wire: The Blog of the Urban Institute. https://www.urban.org/urban-wire/covid-19-crisis-continues-have-uneven-economic-impact-race-and-ethnicity

[bibr16-00027642211003140] Centers for Disease Control and Prevention. (2020). COVID-19 hospitalization and death by race/ethnicity. https://www.cdc.gov/coronavirus/2019-ncov/covid-data/investigations-discovery/hospitalization-death-by-race-ethnicity.html

[bibr17-00027642211003140] Chazan G. (2020, June 4). How Germany got coronavirus right. Financial Times. https://www.ft.com/content/cc1f650a-91c0-4e1f-b990-ee8ceb5339ea

[bibr18-00027642211003140] Chung H. Thewissen S. (2011). Falling back on old habits? A comparison of the social and unemployment crisis reactive policy strategies in Germany, the UK and Sweden. Social Policy & Administration, 45(4), 354-370. 10.1111/j.1467-9515.2011.00779.x

[bibr19-00027642211003140] Collins C. (2019). Making motherhood work: How women manage careers and caregiving. Princeton University Press.

[bibr20-00027642211003140] Collins C. (2020). Who to blame and how to solve it: Mothers’ perceptions of work-family conflict across Western policy regimes. Journal of Marriage and Family, 82(3), 849-874. 10.1111/jomf.12643

[bibr21-00027642211003140] Collins C. Landivar L. C. Ruppanner L. Scarborough W. J. (2020). COVID-19 and the gender gap in work hours. Gender, Work & Organization, 28(Suppl. 1), 101-112. 10.1111/gwao.12506 PMC7361447 32837019

[bibr22-00027642211003140] Collins J. L. Mayer V. (2010). Both hands tied: Welfare reform and the race to the bottom in the low-wage labor market. University of Chicago Press.

[bibr23-00027642211003140] Connell R. W. (1987). Gender and power: Society, the person and sexual politics. Wiley.

[bibr24-00027642211003140] Cook R. Grimshaw D. (2020). A gendered lens on COVID-19 employment and social policies in Europe. European Societies, 23(Suppl. 1). S215-S227. 10.1080/14616696.2020.1822538

[bibr25-00027642211003140] Coronavirus: Denmark lets young children return to school. (2020, April 15). BBC News. https://www.bbc.com/news/world-europe-52291326#:~:text=Children%20up%20to%20the%20age,relax%20coronavirus%20restrictions%20on%20education

[bibr26-00027642211003140] Craig L. Churchill B. (2020). Dual-earner parent couples’ work and care during COVID-19. Gender, Work & Organization, 28(Suppl. 1), 66-79. 10.1111/gwao.12497 PMC7362071 32837023

[bibr27-00027642211003140] Dalley G. (1988). Ideologies of caring: Rethinking community and collectivism. Macmillan.

[bibr28-00027642211003140] Dämmrich J. Blossfeld H.-P. (2017). Women’s disadvantage in holding supervisory positions: Variations among European countries and the role of horizontal gender segregation. Acta Sociologica, 60(3), 262-282. 10.1177/0001699316675022

[bibr29-00027642211003140] Denmark.dk. (2020). Trust: A cornerstone of Danish culture. https://denmark.dk/people-and-culture/trust

[bibr30-00027642211003140] Dubay L. Aarons J. Brown K. S. Kenney G. M. (2020). How risk of exposure to the Coronavirus at work varies by race and ethnicity. Urban Institute. https://www.urban.org/sites/default/files/publication/103278/how-risk-of-exposure-to-the-coronavirus-at-work-varies.pdf

[bibr31-00027642211003140] Eddy M. (2020, December 13). Germany locks down ahead of Christmas as coronavirus deaths rise. The New York Times. https://www.nytimes.com/2020/12/13/world/europe/germany-lockdown-christmas-covid.html

[bibr32-00027642211003140] Elabdi F. (2020, April 16). As Danish schools reopen, some worried parents are keeping their children home. The Washington Post. https://www.washingtonpost.com/world/europe/as-danish-schools-reopen-some-worried-parents-are-keeping-their-children-home/2020/04/16/751eb19e-7f38-11ea-84c2-0792d8591911_story.html

[bibr33-00027642211003140] Esping-Andersen G. (1990). The three worlds of welfare capitalism. Princeton University Press.

[bibr34-00027642211003140] Esping-Andersen G. (1999). Social foundations of postindustrial economies. Oxford University Press.

[bibr35-00027642211003140] Farr C. (2020, July 21). Germany’s coronavirus response is a master class in science communication. CNBC. https://www.cnbc.com/2020/07/21/germanys-coronavirus-response-masterful-science-communication.html

[bibr36-00027642211003140] Ferguson D. (2020, May 3). “I feel like a 1950s housewife”: How lockdown has exposed the gender divide. The Guardian. https://www.theguardian.com/world/2020/may/03/i-feel-like-a-1950s-housewife-how-lockdown-has-exposed-the-gender-divide

[bibr37-00027642211003140] Forde K. (2020, October 6). Mom burnout: Pandemic driving millions of women from us workforce. AlJazeera. https://www.aljazeera.com/economy/2020/10/6/mom-burnout-pandemic-driving-millions-of-women-from-us-workforce

[bibr38-00027642211003140] Germany tears up fiscal rule book to counter coronavirus pandemic. (2020, March 21). Financial Times. https://www.ft.com/content/dacd2ac6-6b5f-11ea-89df-41bea055720b

[bibr39-00027642211003140] Glass J. (2009). Work-life policies: Directions for future research. In Booth A. Crouter N. (Eds.), Work-life policies that make a difference (pp. 231-250). Sage.

[bibr40-00027642211003140] Glass J. Simon R. W. Andersson M. A. (2016). Parenthood and happiness: Effects of work-family reconciliation policies in 22 OECD countries. American Journal of Sociology, 122(3), 886-929. 10.1086/688892 PMC5222535 28082749

[bibr41-00027642211003140] Goger A. Loh T. H. Bateman N. (2020, May 12). Debunking myths about COVID-19 relief’s “unemployment insurance on steroids.” Brookings. https://www.brookings.edu/research/debunking-myths-about-covid-19-reliefs-unemployment-insurance-on-steroids/

[bibr42-00027642211003140] Goodnough A. (2020, July 24). C.D.C. calls on schools to reopen, downplaying health risks. The New York Times. https://www.nytimes.com/2020/07/24/health/cdc-schools-coronavirus.html

[bibr43-00027642211003140] Gornick J. C. Meyers M. K. (2003). Families that work: Policies for reconciling parenthood and employment. Russell Sage Foundation.

[bibr44-00027642211003140] Heggeness M. L. (2020). Estimating the immediate impact of the COVID-19 shock on parental attachment to the labor market and the double bind of mothers. Review of Economics of the Household, 18(4), 1053-1078. 10.1007/s11150-020-09514-x PMC7584481 33132792

[bibr45-00027642211003140] Hipp L. Bünning M. (2020). Parenthood as a driver of increased gender inequality during COVID-19? Exploratory evidence from Germany. European Societies, 23(Suppl. 1), S658-S673. https://www.researchgate.net/publication/345006431_Parenthood_as_a_driver_of_increased_gender_inequality_during_COVID-19_Exploratory_evidence_from_Germany_Parenthood_as_a_driver_of_increased_gender_inequality_during_COVID-19_Exploratory_evidence_from_

[bibr46-00027642211003140] Hjálmsdóttir A. Bjarnadóttir V. S. (2020). “I have turned into a foreman here at home”: Families and work-life balance in times of COVID-19 in a gender equality paradise. Gender, Work & Organization. Advance online publication. 10.1111/gwao.12552 PMC7537149 33041540

[bibr47-00027642211003140] Hochschild A. R. (1983). The managed heart: Commercialization of human feeling. University of California Press.

[bibr48-00027642211003140] Hochschild A. R. (1989). The second shift. Viking.

[bibr49-00027642211003140] International Monetary Fund. (2020). Kurzarbeit: Germany’s short-time work benefit. International Monetary Fund.

[bibr50-00027642211003140] Ismiris B. N. (2018, May 11). Denmark has great maternity leave and child care policies: So why aren’t more women advancing? Harvard Business Review. https://hbr.org/2018/05/denmark-has-great-maternity-leave-and-child-care-policies-so-why-arent-more-women-advancing

[bibr51-00027642211003140] Kiess J. Norman L. Temple L. Uba K. (2017). Path dependency and convergence of three worlds of welfare policy during the Great Recession: UK, Germany and Sweden. Journal of International and Comparative Social Policy, 33(1), 1-17. 10.1080/21699763.2017.1281832

[bibr52-00027642211003140] Kitchener C. (2020, July 28). “The mom shame is so real”: There’s no way to win in the pandemic. The Lily. https://www.thelily.com/the-mom-shame-is-so-real-theres-no-way-to-win-in-the-pandemic/

[bibr53-00027642211003140] Landivar L. C. Ruppanner L. Scarborough W. J. Collins C. (2020). Early signs indicate that Covid-19 is exacerbating gender inequality in the labor force. Socius: Sociological Research for a Dynamic World. Advance online publication. 10.1177/2378023120947997 PMC7399570 34192138

[bibr54-00027642211003140] Leitner S. Lessenich S. (2003). Assessing welfare state change: The German social insurance state between reciprocity and solidarity. Journal of Public Policy, 23(3), 325-347. http://www.jstor.org/stable/4007821

[bibr55-00027642211003140] Levitsky S. R. (2014). Caring for our own: Why there is no political demand for new American social welfare rights. Oxford University Press.

[bibr56-00027642211003140] Lozano A. V. (2020, May 2). A curse for most, a “blessing” for some: How unemployed Americans are getting by during pandemic. NBC News. https://www.nbcnews.com/news/us-news/curse-most-blessing-some-how-unemployed-americans-are-getting-during-n1197351

[bibr57-00027642211003140] MacGillis A. (2020, June 3). How Germany saved its workforce from unemployment while spending less per person than the U.S. ProPublica. https://www.propublica.org/article/how-germany-saved-its-workforce-from-unemployment-while-spending-less-per-person-than-the-u-s

[bibr58-00027642211003140] Madsen L. O. (2020, May 4). COVID-19 has killed working parents’ social contract in Denmark. What now? WorkLife HUB. http://worklifehub.com/blog/covid19-killed-working-parents-social-contract-in-denmark

[bibr59-00027642211003140] Mayrl D. Quinn S. (2016). Defining the state from within: Boundaries, schemas, and associational policymaking. Sociological Theory, 34(1), 1-26. 10.1177/0735275116632557

[bibr60-00027642211003140] Meckler L. Weiner R. (2020, July 24). CDC director concedes schools in “hot spots” face tougher call on reopening. The Washington Post. https://www.washingtonpost.com/education/cdc-director-concedes-schools-in-hot-spots-face-tougher-call-on-reopening/2020/07/24/273ee068-cdd8-11ea-b0e3-d55bda07d66a_story.html

[bibr61-00027642211003140] Miller C. C. (2020, September 17). Private tutors, pop-up schools or nothing at all: How employers are helping parents. The New York Times. https://www.nytimes.com/2020/09/17/upshot/pandemic-workers-benefits-disparity.html

[bibr62-00027642211003140] Miller C. C. Tankersley J. (2020, May 8). Paid leave law tries to help millions in crisis: Many haven’t heard of it. The New York Times. https://www.nytimes.com/2020/05/08/upshot/virus-paid-leave-pandemic.html

[bibr63-00027642211003140] Naumann E. Möhring K. Reifenscheid M. Wenz A. Rettig T. Lehrer R. Krieger U. Juhl S. Friedel S. Fikel M. Cornesse C. Blom A. G. (2020). COVID-19 policies in Germany and their social, political, and psychological consequences. European Policy Analysis, 6(2), 191-202. 10.1002/epa2.1091 PMC7537296 34616900

[bibr64-00027642211003140] Norton M. (2014). Classification and coercion: The destruction of piracy in the English maritime system. American Journal of Sociology, 119(6), 1537-1575. 10.1086/676041

[bibr65-00027642211003140] Novoa C. (2020). How child care disruptions hurt parents of color most. Center for American Progress. https://www.americanprogress.org/issues/early-childhood/news/2020/06/29/486977/child-care-disruptions-hurt-parents-color/

[bibr66-00027642211003140] Oduncu F. S. (2013). Priority-setting, rationing and cost-effectiveness in the German health care system. Medicine, Health Care and Philosophy, 16(3), 327-339. 10.1007/s11019-012-9423-7 22692518

[bibr67-00027642211003140] OECD. (2017). Social security contributions and consumption taxes give way to personal income taxes, as corporate income taxes fail to recover. Author. http://www.oecd.org/newsroom/social-security-contributions-and-consumption-taxes-give-way-to-personal-income-taxes-as-corporate-income-taxes-fail-to-recover.htm

[bibr68-00027642211003140] OECD. (2018). LMF2.1: Usual working hours per week by gender. OECD Family Database. http://www.oecd.org/els/family/LMF_2_1_Usual_working_hours_gender.pdf

[bibr69-00027642211003140] OECD. (2020a). Better Life Index. Author. http://www.oecdbetterlifeindex.org/

[bibr70-00027642211003140] OECD. (2020b). Time use: Time spent in unpaid work, by sex. OECD.Stat. https://stats.oecd.org/index.aspx?queryid=54757

[bibr71-00027642211003140] Olagnier D. Mogensen T. H. (2020). The COVID-19 pandemic in Denmark: Big lessons from a small country. Cytokine & Growth Factor Reviews, 53(June), 10-12. 10.1016/j.cytogfr.2020.05.005 32405247 PMC7217796

[bibr72-00027642211003140] Orloff A. (1993). Gender and the social rights of citizenship: The comparative analysis of gender relations and welfare states. American Sociological Review, 58(3), 303-328. 10.2307/2095903

[bibr73-00027642211003140] Orloff A. (1996). Gender in the welfare state. Annual Review of Sociology, 22(1), 51-78. 10.1146/annurev.soc.22.1.51

[bibr74-00027642211003140] Ornston D. (2020, June 26). Learning from Denmark’s socially inclusive approach to COVID-19. Policy Options. https://policyoptions.irpp.org/magazines/june-2020/learning-from-denmarks-socially-inclusive-approach-to-covid/

[bibr75-00027642211003140] Petts R. J. Carlson D. L. Pepin J. R. (2020). A gendered pandemic: Childcare, homeschooling, and parents’ employment during COVID-19. Gender, Work & Organization. Advance online publication. 10.1111/gwao.12614

[bibr76-00027642211003140] Pew Research Center. (2020a). Fewer mothers and fathers in U.S. are working due to COVID-19 downturn; those at work have cut hours. https://www.pewresearch.org/fact-tank/2020/10/22/fewer-mothers-and-fathers-in-u-s-are-working-due-to-covid-19-downturn-those-at-work-have-cut-hours/

[bibr77-00027642211003140] Pew Research Center. (2020b). How people in 14 countries view the state of the world in 2020. https://www.pewresearch.org/fact-tank/2020/09/23/how-people-in-14-countries-view-the-state-of-the-world-in-2020/

[bibr78-00027642211003140] Pierson P. (1993). When effect becomes cause: Policy feedback and political change. World Politics, 45(4), 595-628. 10.2307/2950710

[bibr79-00027642211003140] Pierson P. (2000). Three worlds of welfare state research. Comparative Political Studies, 33(6-7), 791-821. 10.1177/001041400003300605

[bibr80-00027642211003140] Powell C. (2020). The color and gender of COVID: Essential workers, not disposable people. Think Global Health. https://www.thinkglobalhealth.org/article/color-and-gender-covid-essential-workers-not-disposable-people

[bibr81-00027642211003140] Qian Y. Fuller S. (2020). COVID-19 and the gender employment gap among parents of young children. Canadian Public Policy, 46(Suppl. 2), S89-S101. 10.3138/cpp.2020-077 PMC8230634 38629993

[bibr82-00027642211003140] Rao A. H. (2020). Crunch time: How married couples confront unemployment. University of California Press.

[bibr83-00027642211003140] Redlener I. Sachs J. Hansen S. Hupert N. (2020). Avoidable COVID-19 Deaths—And Counting(2020). 130,000–210,000 —In the U.S (p. 1562). National Center for Disaster Preparedness. https://ncdp.columbia.edu/custom-content/uploads/2020/10/Avoidable-COVID-19-Deaths-US-NCDP.pdf?sid=5c057b533f92a46459c66782&utm_source=newsletter&utm_medium=email&utm_campaign=ctinsider_coronavirus

[bibr84-00027642211003140] Reichelt M. Makovi K. Sargsyan A. (2020). The impact of COVID-19 on gender inequality in the labor market and gender-role attitudes. European Societies, 23(Suppl. 1), S228-S245. 10.1080/14616696.2020.1823010

[bibr85-00027642211003140] Rein L. (2020, April 14). Donald J. Trump’s name will be on stimulus checks in unprecedented move. The Washington Post. https://www.washingtonpost.com/politics/coming-to-your-1200-relief-check-donald-j-trumps-name/2020/04/14/071016c2-7e82-11ea-8013-1b6da0e4a2b7_story.html

[bibr86-00027642211003140] Reuters Staff. (2020, June 4). America’s billionaire wealth jumps by over half a trillion during COVID-19 pandemic. Reuters. https://www.reuters.com/article/us-health-coronavirus-billionaires-idUSKBN23B2SW

[bibr87-00027642211003140] Rosenfeld R. A. Trappe H. Gornick J. C. (2004). Gender and work in Germany: Before and after reunification. Annual Review of Sociology, 30(1), 103-124. 10.1146/annurev.soc.30.012703.110531

[bibr88-00027642211003140] Rugaber C. D’Innocenzio A. (2020, August 14). US retail sales rise for 3rd month but slowdown expected. AP NEWS. https://apnews.com/article/ap-top-news-business-retail-sales-u-s-news-virus-outbreak-44041da6f74749d78c7ea353543f3c9d

[bibr89-00027642211003140] Sachweh P. (2019). Crisis experiences and welfare attitudes during the Great Recession: A comparative study on the UK, Germany and Sweden. Acta Sociologica, 62(2), 135-151. 10.1177/0001699318764261

[bibr90-00027642211003140] Schanzenbach D. W. Pitts A. (2020). How much has food insecurity risen? Evidence from the Census Household Pulse Survey. Institute for Policy Research Rapid Research Report. https://www.ipr.northwestern.edu/documents/reports/ipr-rapid-researchreports-pulse-hh-data-10-june-2020.pdf

[bibr91-00027642211003140] Schmidt M. (2020, September 22). Are women paying the price for COVID-19? DW. https://www.dw.com/en/are-women-paying-the-price-for-covid-19/av-55000113

[bibr92-00027642211003140] Scruggs L. A. Allan J. P. (2008). Social stratification and welfare regimes for the twenty-first century: Revisiting the three worlds of welfare capitalism. World Politics, 60(4), 642-664. 10.1353/wp.0.0020

[bibr93-00027642211003140] Soss J. (2005). Making clients and citizens: Welfare policy as a source of status, belief, and action. In Schneider A. Ingram H. (Eds.), Deserving and entitled: Social constructions and public policy (pp. 291-328). SUNY Press.

[bibr94-00027642211003140] Stelzenmüller C. Denney S. (2020, June 16). Reopening the world: Reopening Germany. Brookings Institute. https://www.brookings.edu/blog/order-from-chaos/2020/06/16/reopening-the-world-reopening-germany/

[bibr95-00027642211003140] Strauss V. (2020, July 25). Confused by CDC’s changing guidance on school reopening? Here are recommendations from experts not pressured by the White House. The Washington Post. https://www.washingtonpost.com/education/2020/07/25/confused-by-cdcs-changing-guidance-school-reopening-here-are-recommendations-experts-not-pressured-by-white-house/

[bibr96-00027642211003140] Thompson D. (2020, March 24). Do more—fast. Don’t wait. The Atlantic. https://www.theatlantic.com/ideas/archive/2020/03/denmark-has-a-message-for-america-do-more-fast/608629/

[bibr97-00027642211003140] U.S. Bureau of Labor Statistics. (2020). Civilian labor force participation rate by age, sex, race, and ethnicity. https://www.bls.gov/emp/tables/civilian-labor-force-participation-rate.htm

[bibr98-00027642211003140] Wiking M. (2016, January 20). Why Danes happily pay high rates of taxes. US News and World Report. https://www.usnews.com/news/best-countries/articles/2016-01-20/why-danes-happily-pay-high-rates-of-taxes

[bibr99-00027642211003140] Williams J. (2000). Unbending gender: Why family and work conflict and what to do about it. Oxford University Press.

